# Simple Method of Arresting Obstinate Epistaxis

**Published:** 1871-01

**Authors:** 


Simple Method of Arresting Obstinate Epistaxis — A writer in the *Gazette des Hopitaux* states that attentive observations of the face of a patient attacked with epistaxis will detect a slight intermittent movement of the soft parts near the ala of the nose on the side where the blood is flowing; even if this pulsation be not seen, it may be felt. Pressure with the finger over this branch of the facial artery is said to arrest the hemorrhage immediately.—*Med. Gazette.*

